# Haematological parameters and muscle oxygen saturation in elite male speed skaters during a 2-min time trial

**DOI:** 10.1007/s00421-025-05955-3

**Published:** 2025-10-09

**Authors:** Jadwiga Malczewska-Lenczowska, Andrzej Klusiewicz, Kinga Rębiś, Adam Czaplicki, Tomasz Kowalski, Dominika Granda, Michał Starczewski, Marcin Konopka

**Affiliations:** 1 Department of Nutrition Physiology, Institute of Sport, National Research Institute, Warsaw, Poland; 2 Department of Physiology and Biochemistry, Faculty of Physical Education and Health in Biała Podlaska, Józef Piłsudski University of Physical Education, Warsaw, Poland; 3 Department of Physiology, Institute of Sport, National Research Institute, Warsaw, Poland; 4 Department of Biomechanics and Anatomy, Faculty of Physical Education and Health in Biała Podlaska, Józef Piłsudski University of Physical Education, Warsaw, Poland; 5 Department of Physiotherapy Fundamentals, Faculty of Rehabilitation, Józef Piłsudski University of Physical Education, Warsaw, Poland; 6 Department of Sports Cardiology and Noninvasive Cardiovascular Imaging, Faculty of Medicine, Medical University of Warsaw, Warsaw, Poland

**Keywords:** Cycling time trial, Speed skating, tHb_mass_, Intravascular volume indices, Blood count indices, Near-infrared spectroscopy-derived muscle oxygen saturation, Vastus lateralis muscle

## Abstract

**Purpose:**

This study analysed the relationship between haematological indices and near-infrared spectroscopy (NIRS) parameters, as they are rarely explored.

**Methods:**

Nineteen elite male speed skaters performed a 2-min cycling time trial (TT) during which muscle oxygen saturation (SmO_2_) and local haemoglobin concentration (tHb_muscle_) were continuously monitored using NIRS. Haematological parameters was evaluated through (1) total haemoglobin mass (tHb_mass_), plasma volume (PV), blood volume (BV), and red cell volume (RCV), using the carbon monoxide (CO) rebreathing method, and (2) complete blood count from venous blood samples. Exploratory correlation and regression analyses were conducted to investigate potential associations between the variables.

**Results:**

Baseline SmO_2_ was not associated with any parameters, whereas tHb_muscle_ was positively correlated with RCV, BV, and PV (*p* < 0.05). SmO₂ values exhibited three distinct phases: a descent and plateau during the TT, followed by an ascent during recovery. None of the haematological variables explained SmO_2_ variability in the entire cohort. After dividing the participants based on the median of haematological variables into *low* and *high* groups, regression analyses showed that dichotomised tHb_mass_ (*p* < 0.01), BV (*p* < 0.01), and PV (*p* < 0.05) explained the variability of SmO_2_ only in the linear part of the descent phase. During recovery, half-time of local muscle reoxygenation correlated negatively with relative values of PV, BV (*p* < 0.05) and tHb_mass_ (*p* < 0.06).

**Conclusions:**

Haematological status, irrespective of the assessment method, did not appear to influence either resting SmO_2_ or SmO₂ kinetics during the time trial. However, muscle reoxygenation dynamics may be associated with haematological parameters obtained via the CO rebreathing method.

## Introduction

Oxygen transport to tissues is considered one of the main factors limiting the body's aerobic capacity in well-trained athletes (Bassett and Howley 2000; González-Alonso and Calbet 2003). The efficiency of this process depends on the performance of the circulatory system (stroke volume, cardiac output, and blood volume), vascular adaptation (e.g. structure and function of conduit and resistant arteries) (Green et al. 2012), as well as the oxygen-carrying capacity of arterial blood, which is primarily determined by the amount of haemoglobin (Bassett and Howley 2000; Joyner and Casey 2015) and blood volume (BV) (Webb et al. 2023). According to some authors, in healthy individuals, BV appears to be the primary regulator of oxygen transport to the exercising muscles (Gledhill et al. 1999; Schmidt and Prommer 2008). On the one hand, an increase in BV may enhance overall blood flow and improve oxygen delivery to tissues (Sawka et al. 2000; Zouhal et al. 2023). Notably, BV expansion due to increased plasma volume typically leads to a reduction in haemoglobin concentration (i.e. less haemoglobin per unit volume of blood), which could compromise optimal oxygen transport and result in an inverse relationship between blood volume and tissue oxygenation (Schierbauer et al. 2024). However, a sustained state of haemodilution, as well as physical exertion itself, may stimulate the activation of erythropoiesis, ultimately leading to its increase to restore appropriate Hb levels (Schwandt et al. 1991).)

In competitive athletes, especially those participating in endurance sports, the total amount of haemoglobin in the blood is substantially higher than in other sports and in untrained individuals and recreational athletes (Heinicke et al. 2001), while the concentration of haemoglobin [Hb] does not differ and sometimes is even lower in athletes (Heinicke et al. 2001; Malczewska-Lenczowska et al. 2013). The reason for this phenomenon is the body's adaptations, leading to haemodilution, primarily through an increase in plasma volume (Sawka et al. 2000; Schmidt and Prommer 2008; Zouhal et al. 2023). Therefore, [Hb] does not always reflect the total haemoglobin amount in the body (Malczewska-Lenczowska et al. 2013; Schierbauer et al. 2024).

Total haemoglobin mass (tHb_mass_) is the key indicator that allows for the direct determination of the total amount of haemoglobin (in grams) in the entire bloodstream. Importantly, tHb_mass_ is independent of plasma volume (PV), meaning that changes in plasma volume do not affect its value (Schmidt and Prommer 2005, 2008). Moreover, scientific research shows that tHb_mass_ and blood volume (BV) exhibit stronger correlations with endurance performance predictors, such as *V*O₂max and maximal or submaximal power output, than [Hb] (Heinicke et al. 2001; Schmidt and Prommer 2008; Jacobs et al. 2011). Increased oxygen demand during exertion triggers not only systemic responses, such as increased cardiac output, but also local reactions, including increased blood flow in working muscles (Crispin 2020). Currently, the observation of muscle oxygen saturation (SmO₂) during exercise at the microvascular level is possible using near-infrared spectroscopy (NIRS) (Hamaoka et al. 2007; Ferrari et al. 2011), which typically relies on the oxygen-dependent properties of light in the 650–950 nm range. SmO_2_ is recognized as a more reliable indicator of physical performance and fatigue than arterial oxygenation alone (Barstow 2019). During physical exercise, changes in deoxy- and oxy-haemoglobin and myoglobin signals provide information on the degree of tissue oxygen saturation and reflect the balance between oxygen supply and its consumption in muscles (Boushel et al. 2001; Buchheit and Ufland 2011). Muscle total haemoglobin (tHb_muscle_), in turn, reflects the local haemoglobin content in the blood perfusing the working muscle at the time of measurement. Since the tHb_mass_ in the body generally does not change during physical exertion, variations in tHb_muscle_ during exercise are interpreted as changes in local blood volume within the microvascular compartment (Alvares et al. 2020).

Microvascular blood flow in skeletal muscle is influenced by multiple factors, including the type and intensity of exercise, training status, and various local regulatory mechanisms (González-Alonso and Calbet 2003; González-Alonso et al. 2006; Perrey et al. 2024; Izumi et al. 2025).

Over the last two decades, studies on the response of SmO_2_ to various physical exercises, measured non-invasively using NIRS at different body sites, have expanded the knowledge on the mechanisms underlying the oxygen uptake response (Perrey and Ferrari 2018). However, the changes in muscle tissue oxygenation, metabolism and haemodynamics in response to specific exercise stimuli are still a poorly understood area of exercise physiology (Perrey et al. 2024). Furthermore, the relationship between the body's haematological status and tissue oxygenation is relatively underexplored (Gandia-Soriano et al. 2022), and the results are not unequivocal (González-Alonso et al. 2006; Alvares et al. 2020; Gandia-Soriano et al. 2022). Crispin and Forwood (2021), when discussing this issue, emphasized that although some studies have shown a positive correlation between haemoglobin concentration [Hb] and tissue SmO_2_, improvements in tissue oxygenation with increasing [Hb] were observed mainly in brain areas, not in muscle tissue. The lack of such relationships could be influenced by the limitations of [Hb] as an indicator of the amount of haemoglobin in the body (Malczewska-Lenczowska et al. 2013; Schierbauer et al. 2024). Although the literature underlines the importance of haematological adaptations in improving endurance capacity (Schmidt and Prommer 2008; Montero et al. 2015), there are no studies addressing the impact of haematological status on SmO_2_ and tHb_muscle_, and their kinetics at rest and during exercise. The aim of this study was to analyse the relationship between the total amount of haemoglobin in the body, assessed both by blood morphology indices and tHb_mass_ including intravascular volume indices (plasma, blood and red cell volumes), and SmO_2_ and tHb_muscle_ at rest, during a 2-min cycling time trial (TT), and during the recovery period.

## Material and methods

### Study design

The study was conducted using a cross-sectional design. The examinations were carried out over two consecutive days. On the first day, fasting venous blood samples were collected in the morning. After breakfast, participants performed a 2-min cycling time trial (TT). Subsequently, total haemoglobin mass (tHb_mass_) was measured no earlier than 2 h after the exercise test to minimize the potential influence of exercise on the measurement. The reason for this is that increased muscle blood flow and greater capillary surface area during exercise both increase the rate of CO diffusion to intramuscular myoglobin and lead to higher calculated Hb_mass_ values (Schmidt et al. 2003). This process can continue for several minutes after exercise, so we have adhered to a safety period of 2 h in accordance with the recommendation of Blood tec. This time interval allows for the restoration of physiological conditions and ensures comparable carbon monoxide diffusion in the body (Gough et al. 2012; Schmidt et al. 2023) On the 2nd day, fasting venous blood samples were again collected (this time for blood count analysis), anthropometric measurements were performed, and after breakfast, participants performed the tHb_mass_ measurement for the second time. The experimental protocol was approved by the Ethics Committee of the Institute of Sport–National Research Institute (KEBN-22-76-JM; 2022.12.10). Written informed consent was obtained from the participants. This study was carried out in accordance with the Declaration of Helsinki (2000) of the World Medical Association.

### Participants

Initially, 20 elite male speed skaters took part in the study; one of them was excluded from the analysis due to data recording errors. All included participants (*n* = 19) were members of the national junior and senior teams, trained in the same group with a similar exercise programme, and were in the same training phase (early preparatory period). The athletes were considered eligible for testing after taking into account the exclusion criteria (apparent signs of acute or chronic illness, latent iron deficiency and iron deficiency anaemia, as well as recent skeletal muscle injury that could affect the assessment of muscle oxygen saturation and performance of physical effort). As a result, at the time of the study, all participants were in good health, non-smokers, and unmedicated. During the 24 h preceding the study, all skaters were asked to refrain from caffeine, alcohol, and vigorous exercise. All participants were Caucasian, they were informed of the study design and the physical tasks ahead of time, and written informed consent was obtained in advance. General characteristics of the examined male speed skaters are presented in Table 1.

**Table 1:** Basic anthropometric, training, and haematological and exercise test indices in studied male athletes, *n* = 19

Variable	Outcome (mean ± SD)
Age (years)	20.5 ± 3.72
*Anthropometric*	
Stature (cm)	182.7 ± 6.5
Total body mass (kg)	77.3 ± 7.7
BMI (kg/m^2^)	23.1 ± 1.5
Fat mass (%)	10.0 ± 3.3
ATT in vastus lateralis area (mm)	4.8 ± 1.1 (3.2–6.9)
*Training*	
Training experience (years)	8.5 ± 4.3
Current training volume (hours/week)	22.1 ± 6.5
*Haematological*	
[Hb] (g/dL)	15.1 ± 1.0
Hct (%)	43.6 ± 2.6
RBC (× 10^12^/L)	5.0 ± 0.4
MCH (pg)	30.2 ± 1.1
MCV (fL)	87.4 ± 2.7
rel.tHb_mass_ (g/kg)	12.1 ± 1.0
rel.PV (ml/kg)	53.2 ± 5.3
rel.BV (ml/kg)	88.4 ± 7.5
*Pre-exercise NIRS indices*	
SmO_2_ (%)	64.4 ± 7.6
tHb_muscle_ (g/dl)	12.9 ± 0.22
*Exercise muscle oxygen saturation*	
SmO_2min_ (%)	9.4 ± 4.2
SmO_2max (%)_	78.3 ± 3.1
ΔSmO_2_ (%)	68.8 ± 5.5
*Cycling time-trial performance*	
*P*_mean_ (W)	512 ± 61.4
rel.*P*_mean_ (W/kg)	6.62 ± 0.63
Cycled distance (m)	1731 ± 74
La3min (mmol/L)	15.6 ± 2.2

*ATT* skinfold thickness, *BMI* body mass index, *[Hb]* blood haemoglobin concentration, *Hct* haematocrit, *RBC* red blood cells, *MCV* mean corpuscular volume (red cell volume), *MCH* mean corpuscular haemoglobin, *rel.tHb*_*mass*_ total haemoglobin mass/kg body mass, *rel.PV* plasma volume expressed per kg body mass, *rel.BV* blood volume expressed per kg body mass, *SmO*_*2*_ muscle oxygen saturation, *SmO*_*2min*_ minimum local muscle oxygen saturation during 2-min time trial, *SmO*_*2max*_ maximum local muscle oxygen saturation during 2-min time trial, *ΔSmO*_*2*_ difference in maximum and minimum muscle oxygen saturation during exercise, *tHb*_*muscle*_ muscle local haemoglobin concentration, *P*_*mean*_ mean power, *rel.P*_*mean*_ mean power expressed per kg body mass, *La3min* lactic acid concentration at 3 min after exercise test

### Experimental procedures

#### Blood analysis

Blood sample collection from the antecubital vein started after 15 min of rest in a seated position to eliminate any residual effect of physical movement on plasma volume and ensure that the data collected reflected a resting baseline. The following indices were determined in whole blood using a haematology analyser (Sysmex 1000x, Japan): [Hb], haematocrit (Hct), red blood cell count (RBC), mean corpuscular volume (MCV), and mean corpuscular haemoglobin (MCH).

To assess iron status and to exclude an acute phase reaction, the following indices were determined: ferritin, soluble transferrin receptor, and C-reactive protein concentration (CRP) in serum (immunoturbidimetric method, Cobas Integra 400Plus, Roche, ABX, Switzerland), and white blood cell count (WBC) (haematology analyser Sysmex 1000x, Japan), as well as erythrocyte sedimentation rate (ESR) after 1 h in whole blood samples. All blood analyses were performed in a laboratory with an implemented quality system at the Institute of Sport-National Research Institute (protocol of accreditation #AB946).

#### Cycling time-trial (TT) performance

The male skaters performed a 2-min TT on the Wattbike cycle ergometer (Wattbike Ltd, Nottingham, UK). The TT duration was established to simulate a classic 1500 m race on ice, as it takes a similar time and corresponds to the participants’ racing requirements. The effort was self-paced, and the participants were instructed to maintain the highest sustainable intensity throughout the whole test. All participants were familiar with the setting, as they had performed multiple training and testing sessions with the Wattbike cycle ergometer before. The ergometer resistance and cycling cadence were self-selected. Exercise tests were performed under similar environmental conditions (ambient temperature 20–22 °C and relative humidity 40–60%). The following exercise variables were measured and calculated: absolute and relative mean power output (*P*_mean_), cycled distance (m), and capillary blood lactate concentration at 3 min after exercise.

#### Measurements of muscle oxygen saturation (SmO_2_) and local total haemoglobin (tHb_muscle_) in vastus lateralis (VL)

The NIRS device (Moxy Monitor; Fortiori Design LLC, Hutchinson, MN, USA) was placed on the VL muscle, which is the major contributor to power generation in cycle ergometry (Okushima et al. 2016). The Moxy monitor is a continuous wave near-infrared spectroscopy monitor. It uses a new type of algorithm that is based on Monte Carlo modelling, which includes the effects of an unknown adipose tissue thickness (ATT) by modelling a range of ATT (Feldmann et al. 2019). The system uses four wavelengths: 680, 720, 760, and 800 nm. It has two emitters to detector spacings of 12.5 and 25 mm. The device was placed approximately 15 ± 2 cm above the proximal border of the patella on the vastus lateralis muscle belly and fixed to the right limb with a dark 7.5 cm dynamic tape by the same person.

Muscle oxygen saturation variables, i.e. percentage of SmO_2_ and tHb_muscle_, were measured continuously at baseline (starting 3 min after the warm-up), during the TT, and during 3 min of recovery. After warm-up and during recovery the measurements were performed while the athletes remained in a sitting position with the lower limbs in a fixed position. The SmO_2_ data values for further calculations were averaged from measurements (every 2 s) obtained during 120 s, 3 min after warm-up, directly before tests (SmO_2baseline_), during exercise to capture the minimum and maximum value of muscle oxygen saturation (SmO_2min_ and SmO_2max_, respectively), as well as to assess ΔSmO_2_ and during recovery to estimate the half-time of local muscle reoxygenation (t½SmO_2_). The mean SmO_2_ for 2 s immediately following the completion of the exercise was defined as 0% and the peak SmO_2_ in the first 3 min of the recovery phase was defined as 100%. t½SmO_2_ was then defined as the time from the completion of exercise to the point at which 50% of the post-exercise SmO₂ peak was reached (McCully et al. 1994; Nagasawa 2013). tHb_muscle_ was recorded within the same period and sampling frequency. The skinfold tissue thickness (ATT) was measured twice exactly at the site of NIRS measurement (over the vastus lateralis muscle) with a skinfold calliper (Harpenden, Baty International, Burgess Hill, West Sussex, UK) directly before 2-min TT by an expert in anthropometry. The study by Feldmann et al. (2019) using the Moxy Monitor showed that caution is warranted with ATT > 15 mm. In our sample, the mean value of ATT was 4.8 ± 1.1 mm, and the highest value did not exceed 6.9 mm. Thus, the adipose tissue thickness in the VL area should not have interfered with the NIRS signal.

#### Assessment of total haemoglobin mass (tHb_mass_) and intravascular volume indicators.

tHb_mass_ was determined by applying an optimized carbon monoxide rebreathing method according to Schmidt and Prommer (2005). This measurement allows for quantitative assessment (in grams) of total amount of Hb in the bloodstream as well as volume parameters, i.e. total blood, plasma and erythrocyte volumes. All measurements were taken by the same two highly experienced technicians. The examination was started always after resting for at least 15 min in a seated position. During the measurement, the participants breathed for 2 min a mixture of about 4 L of pure medical oxygen (99.5% O_2_) with an individual dose, depending on body mass (1.0 ml·kg^−1^ BM) of carbon monoxide (CO, 4,7 Linde AG, Germany). A CO sensor (Pac 7000, Dräger, Germany) was used to check potential leaks of gas during the measurement and CO content in exhaled air before and after the measurement, as well as to measure the remaining CO content in the bag after the procedure. Before blood collection, a warming ointment with capsaicin (Finalgon, Boehringer Ingelheim, Germany) was applied to the earlobe to increase blood flow. Capillary blood (~ 100 µl) from the earlobe was collected into heparinized tubes three times: just before the measurement (3 samples), and in the 6th and 8th min (2 samples), counting from the beginning of the measurement. The percentage value of carboxyhaemoglobin (%HbCO) in the blood was determined using the CO oximeter (ABL90Flex, Radiometer, Denmark). tHb_mass_ was calculated using dedicated software (blood volume measurements: SpiCO; Blood tec, Bayreuth, Germany). Each participant performed the measurement twice. In three cases, when the error of two measurements exceeded the permissible 2% threshold, the tHb_mass_ measurement was performed for the third time, approximately 5 h after the previous measurement. The precision of tHb_mass_ in the examined speed skaters, obtained from double measurements, was 1.67%. Using the values of vein haemoglobin concentration and haematocrit, intravascular volume indices, i.e. plasma volume, blood volume, and red cell volume, were also calculated.

#### Anthropometric measurements

Body mass and body composition were measured using a bioelectrical impedance analysis (BIA) system (Tanita BC-420MA, Japan) to assess fat mass in the preprandial state at 8:00–9.00 a.m. Stature was measured using a stadiometer (Seca 285, Germany).

### Statistical analyses

At the beginning of the analysis, the nature of the distributions of the measured data was examined using Shapiro–Wilk and Henze–Zirkler tests. The first test showed the normal distributions of the measured variables, while the second revealed bivariate normal distributions for all pairs of these variables. Pearson's correlation coefficients were then determined for the relevant variables, and multiple regression analyses were performed taking SmO_2_ as the dependent variable. The calculations used a bidirectional stepwise algorithm, which allowed variables to be added or removed from the model in successive iterations.

**Fig. 1:**
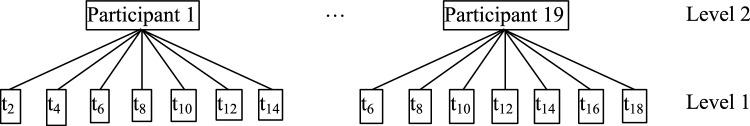
Basic hierarchical model

In the second stage of the statistical analysis, a hierarchical linear mixed model was created, consisting of two levels: Level 1 and Level 2 (Fig. 1). The first level comprised repeated measurements of SmO_2_ conducted at constant 2-s intervals (within-person model), while the second level comprised the participants (between-person model) (Szyszka and Czaplicki 2021; Szyszka et al. 2022).

The basic model was expanded to include a time varying covariate tHb_muscle_ measured simultaneously by the Moxy device. This variable was decomposed into the person-mean-centred time varying covariate (tHb_muscle_.cent) and embedded at Level 1 and the person-mean (tHb_muscle_.mean) attached to Level 2 (Szyszka et al. 2021). Each of the independent haematological variables entering the model was dichotomized relative to the median into Group{high, low} (e.g. rel.tHb_mass_.high) and used as a predictor of SmO_2_ at Level 2. Considering that the subject of modelling was SmO_2_ time series, the model also included the interactions Time*tHb_muscle_.cent and Time*Group. The computations used the *lmer* function from the *lmerTest* package and the *step* function, which performs automatic elimination of statistically insignificant effects from the fixed and random parts of the input model generated by *lmer* (Kuznetsova et al. 2017). The model parameters were estimated using the restricted maximum likelihood (REML) method.

The final form of the model can be expressed as follows:

1 $${SmO}_{{2}_{ij}}={\gamma }_{00}+{\gamma }_{10}{Time}_{ij}+{\gamma }_{11}{Time}_{ij}t{Hb}_{muscle}.{cent}_{ij}+{\gamma }_{12}{Time}_{ij}Group+{u}_{oj}+{u}_{1j}{Time}_{ij}+{r}_{ij},$$

where lower subscripts *i* denote measurement sessions, *j* (1 ÷ 19) participants, *gammas* are regression coefficients, *u*_*0j*_ and *u*_*1j*_ represent random deviations from the overall mean intercept and slope for the participant *j*, and *r*_*ij*_ stands for within-participant residual error. The Group takes the values 0 for the high group and 1 for the low group, as the former was treated as the reference group in the calculations. The first four components on the right-hand side of Eq. 1 define the fixed part of the model, while the remaining three define the random part.

All statistical analyses were conducted in R (R Foundation for Statistical Computing, Austria). The statistical significance of all analyses was set at *p* < 0.05.

## Results

The results of the correlation analysis between mutual quantitative haematological indices (blood red cell indices vs. parameters obtained using the CO rebreathing method) as well as between haematological and NIRS indicators with mean power in 2-min cycling TT are presented in Table 2.

**Table 2:** Correlation analysis between quantitative haematological indices and selected NIRS indicators and mean power in cycling time-trial performance in male speed skaters (*n* = 19)

	SmO_2baseline_	SmO_2max_	SmO_2min_	ΔSmO_2_	t½SmO_2_	tHb_muscle_	*P*_mean_	rel.*P*_mean_	[Hb]	Hct
rel. tHb_mass_	0.18	0.05	0.05	0.00	– 0.45	0.42	0.20	0.54*	0.38	0.50*
rel. RCV	0.16	0.01	0.04	– 0.03	– 0.45	0.52*	0.22	0.57*	0.25	0.46*
rel. PV	0.17	– 0.25	– 0.07	– 0.09	– 0.50*	0.46*	0.39	0.73***	– 0.65**	– 0.52*
rel. BV	0.19	– 0.17	– 0.03	– 0.08	– 0.55*	0.56*	0.37	0.76***	– 0.35	– 0.16
[Hb]	0.00	0.29	0.10	0.09	0.13	– 0.17	– 0.24	– 0.26	–	–
Hct	– 0.01	0.26	0.12	0.06	0.07	0.04	– 0.19	– 0.16	–	–
MCH	0.07	– 0.05	– 0.02	– 0.02	– 0.04	– 0.56*	– 0.14	– 0.38	–	–

*[Hb]* venous haemoglobin concentration, *Hct* haematocrit, *MCH* mean corpuscular haemoglobin in erythrocytes, *rel.tHb*_*mass*_ total haemoglobin mass/kg of body mass, *rel.RCV* red cell volume/kg body mass, *rel.BV* blood volume/kg body mass, *rel.PV* plasma volume/kg body mass, *tHb*_*muscle*_ muscle local haemoglobin concentration before exercise, *SmO*_*2baseline*_ local muscle oxygen saturation before exercise, *SmO*_*2max*_ maximum muscle oxygen saturation during 2-min time trial, *SmO*_*2min*_ minimum muscle oxygen saturation during 2-min time trial, *ΔSmO*_*2*_ difference in maximum and minimum muscle oxygen saturation during exercise, *t½SmO*_*2*_ half-time of muscle reoxygenation, *rel.P*_*mean*_ mean power/kg body mass

Pearson correlation coefficient significance: ^**.**^*p* < 0.1, *p < 0.05, **p < 0.01, ***p < 0.001

### Correlations of haematological indices and pre-exercise NIRS indices

Baseline SmO_2_ was not associated with any haematological parameters, whether measured in blood or determined using the carbon monoxide method. However, the pre-exercise NIRS-derived tHb_muscle_ value showed a moderate positive association with three relative intravascular indices—RCV (*r* = 0.52; *p* < 0.05), BV (*r* = 0.56; p < 0.05), and PV (*r* = 0.46; *p* < 0.05)—as well as a moderate negative association with MCH (*r* = –  0.56; p < 0.05). The correlation between pre-exercise tHb_muscle_ and relative tHb_mass_ was not statistically significant (r = 0.42; p < 0.07).

### Correlations of haematological indices with exercise and recovery NIRS indicators

No haematological parameters were correlated with SmO_2max_, SmO_2min_ or ΔSmO_2_. A significant moderate negative association were observed only between t½SmO_2_ and relative values of blood volume (*r* = – 0.55; *p* < 0.05) and plasma volume (*r* = – 0.50; *p* < 0.05). A similar direction and strength of association between t½SmO_2_ and both relative RCV and relative tHb_mass_ was observed, although the latter did not reach statistical significance (*r* = – 0.45; *p* < 0.05 and *r* = – 0.45; *p* = 0.06, respectively).

### Correlations of haematological indices with mean power in 2-min TT

All four intravascular indices, i.e. relative values of tHb_mass_ (*r* = 0.54; *p* < 0.05), PV (*r* = 0.73; *p* < 0.001), BV (*r* = 0.76; *p* < 0.001), and RCV (*r* = 0.57; *p* < 0.05), showed moderate positive association with the relative value of *P*_mean_. In contrast, no such correlation was observed for [Hb], Hct and MCH. Furthermore, in the subgroup with *high* rel.tHb_mass_, the relative value of *P*_mean_ (6.63 ± 0.51 W/kg) was significantly higher (*p* < 0.05) than in the *low* rel.tHb_mass_ group (6.34 ± 0.56 W/kg) (not shown in tables).

### Correlations between blood count variables and haematological parameters obtained using CO rebreathing method

[Hb] was moderately correlated only with relative plasma volume (*r* = – 0.65; *p* < 0.01), while haematocrit showed a moderate positive correlation with the relative values of tHb_mass_ (*r* = 0.50; *p* < 0.05) and RCV (*r* = 0.46; *p* < 0.05), as well as a moderate negative correlation with the relative value of PV (*r* = – 0.52; *p* < 0.05).

### *Time trajectories of SmO*_*2*_* and tHb*_*muscle*_* changes before, during and after 2-min TT*

Observing the time courses of SmO_2_, three distinct phases can be distinguished at the beginning, during the exercise and the recovery. Specifically, during the initial phase, SmO_2_ exhibited a linear decline (negative slope), followed by a plateau, and subsequently a linear increase (positive slope). The onset of each phases varied from participant to participant.

**Fig. 2:**
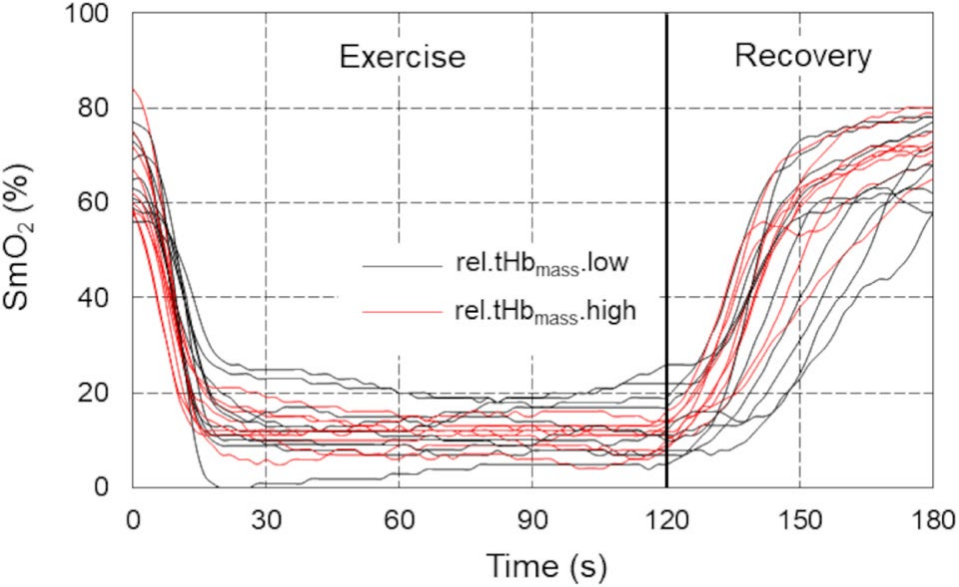
Time courses of the muscle oxygen saturation for individual participants registered before, during, and after exercise 2-min cycling time-trial performance in male speed skaters (*n* = 19). SmO_2_—local muscle oxygen saturation in groups with *low* (*n* = 10, black lines) and *high* (*n* = 9, red lines) relative total haemoglobin mass

Figure 2 shows the measured individual time courses of the oxygen saturation level of the examined muscle during the entire observation period. The linear nature of large portions of the SmO_2_ waveforms in all 3 phases is clearly shown.

**Fig. 3:**
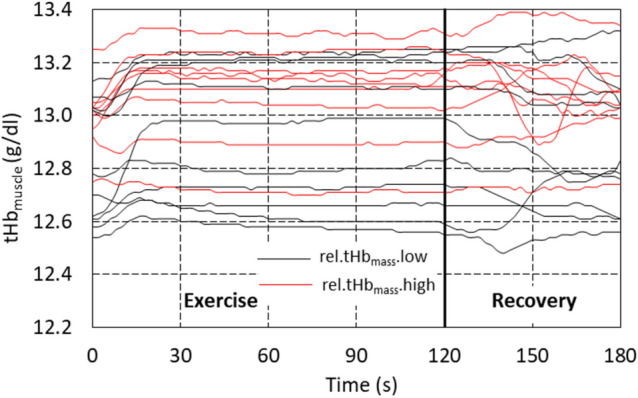
Time courses of tHb_muscle_ in the examined muscle for individual participants recorded by NIRS method before, during and after cycling time-trial performance in male speed skaters (*n* = 19). tHb_muscle_—local muscle haemoglobin concentration in groups with *low* (*n* = 10, black lines) and *high* (*n* = 9, red lines) relative total haemoglobin mass

Figure 3 presents the individual time courses of local muscle total haemoglobin concentration in the examined muscle determined by the NIRS method in groups with *low* and *high* rel.tHb_mass_. The variation of these waveforms in individual participants during phases of descent and recovery SmO_2_ and the approximately constant level of tHb_muscle_ in the plateau phase can again be seen. The increase in tHb_muscle_, meaning an increase in blood flow in the microcapillary system, took place in the phase of SmO_2_ descent in both groups (with *low* and *high* rel.tHb_mass_), and the rate of tHb_muscle_ increase did not differ between groups.

Table 3 shows the results of the regression analysis for the three linear approximations of the recorded SmO_2_ level waveforms of the examined muscle during and after cycling time-trial performance in male speed skaters.

**Table 3:** Estimation of model parameters in the examined male speed skaters (*n* = 19)

	Exercise		Recovery
	Descent phase	Plateau phase	Ascent phase
Variable	Model 1	Model 2	Model 3
	Coefficient (SE)		
Fixed effects			
Intercept (*γ*_*00*_)	75.61^***^ (3.01)	12.03^***^ (1.42)	– 241.24^***^ (18.08)
Slope (*γ*_10_)	– 4.20^***^ (0.31)	– 0.012 (0.016)	2.03^***^ (0.14)
Time*tHb_muscle_.cent (*γ*_11_)	– 2.89^**^ (0.95)	– 0.009 (0.047)	– 0.08^*^ (0.04)
Time*Group (*γ*_12_)	0.79^**^ (0.26)	0.019 (0.018)	– 0.08^∙^ (0.04)
	Variance (SD)		
Random effects			
Level-1 residuals (*rij*)	2.77 (1.66)	0.53 (0.73)	1.74 (1.32)
Level-2 residuals			
Intercept (*u*_*oj*_)	156.50 (12.51)	37.68 (6.14)	5990.46 (77.40)
Time (*u*_*1j*_)	1.22 (1.11)	0.004 (0.062)	0.37 (0.61)
– 2 LL	652.15	2216.77	1036.05

*LL* log likelihood, *descent phase* the period of linear decrease of SmO_2_ at the beginning of exercise (approx. 18 s), *plateau phase* the period of minimal slope of the SmO_2_ time during exercise, *ascent phase* linear increase of SmO_2_ during recovery, *tHb*_*muscle*_*.cent* person-mean-centred value of tHb_muscle_ in each phase, *Group.rel.tHb*_*mass*_*.low* the group with rel.tHb_mass_ below the median value

∙*p* < 0.1, *p < 0.05, **p < 0.01, ***p < 0.001

### Descent phase

**Fig. 4:**
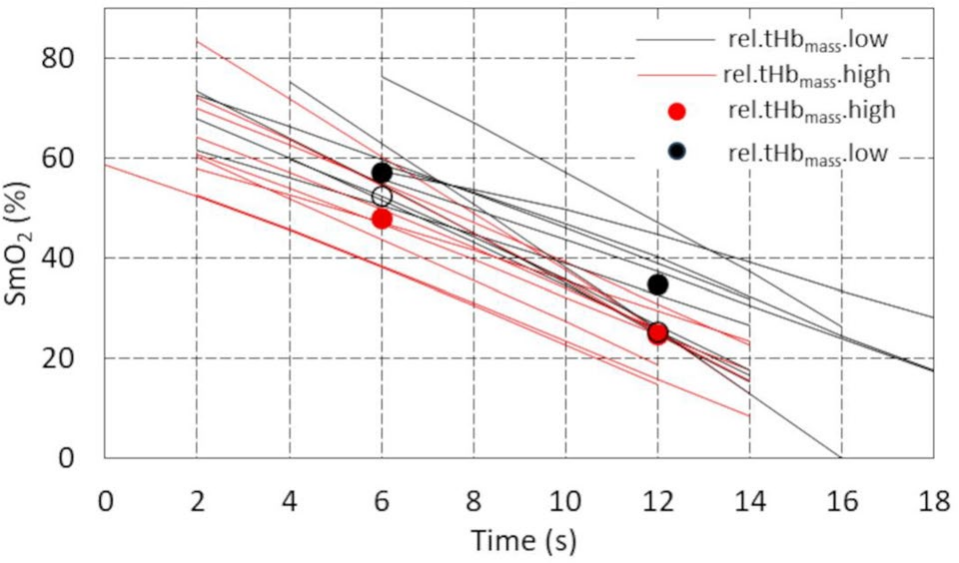
Cross-level interaction Time*Group of muscle oxygen saturation in the decent phase. SmO_2_ – local muscle oxygen saturation in groups with *low* (*n* = 10, black lines) and *high* (*n* = 9, red lines) relative total haemoglobin mass

The average value of the intercept and the rate of SmO_2_ decline for all participants were 75.61% and -4.20%*s^−1^, respectively. The results also showed significant Time*tHb_muscle_.cent (*p* < 0.01) and Time*Group (*p* < 0.01) interactions. The former (a within-person interaction) reflected the individual characteristics of tHb_muscle_.cent waveforms during the descent phase. A positive γ_12_ value attributed to the cross-level interaction (Table 3) means that the difference in SmO_2_ between groups increases by 0.79% every second. The effect of this interaction on SmO_2_ curves is explained in Fig. 4. The difference between the mean SmO_2_ values for the *high* (red dots) and *low* (black dots) groups increased significantly over time, taking values of 9.23% (*p* < 0.05) in the 6th and 10.19% (*p* < 0.01) in the 12th second from the start of TT. If this interaction did not occur, the difference between the *high* and *low* groups (empty dots) would be 4.49% and 0.79%, respectively, and would be statistically insignificant. Although the pre-exercise SmO_2_ level did not differ significantly between the groups (66.1%, and 65.8% in *high* and *low* groups, *p* < 0.94), Fig. 4 also shows that the linear decrease in SmO2 started earlier in the high group.

Very similar results in the descent phase were also obtained with rel.PV (– 2LL = 655.65; Time*Group = 0.57, *p* < 0.05) and rel.BV (– 2LL = 652.60; Time*Group = 0.81, *p* < 0.01) after dichotomising these variables.

In contrast, no associations were found between SmO_2_ and [Hb] (p = 0.75), RBC (*p* = 0.98), Hct (*p* = 0.37), MCH (*p* = 0.23), or the sum of two or more haematological variables.

### Plateau phase

There is a minimal slope of the SmO_2_ time courses in this phase; hence, the value of the intercept corresponds approximately to the mean level of this variable for all participants. The regression coefficients in this phase were found to be statistically non-significant.

### Ascent phase/recovery

The negative value of the intercept in the recovery phase is a consequence of the positive slope of the linearly approximated SmO_2_ waveforms. The mean slope of these waveforms was found to be statistically significant, as was the Time*tHb_muscle_.cent interaction (*p* < 0.5). There was a borderline statistically significant result (*p* < 0.05) for the regression coefficient of the cross-level interaction Time*Group (Table 3).

Finally, it is worth noting that the rate of SmO_2_ increase during recovery was approximately 2 times slower than the decrease during the descent phase (+ 2.03%/s vs −4.2, respectively), and the person-mean tHb_muscle_ was statistically non-significant in all the models we tested.

## Discussion

The primary aim of the study was to determine whether the haematological status of athletes is associated with baseline values and the kinetics of NIRS-derived indices (SmO_2_ and tHb_muscle_) during a standardized 2-min TT and subsequent recovery. The results indicated that haematological status—regardless of the assessment method—does not affect resting skeletal muscle SmO_2_ values, although blood and plasma volumes may influence tHb_muscle_. During the TT, variations in muscle SmO₂ kinetics related to total haemoglobin content were observed, but only during the initial phase of the test.

### *Baseline values of SmO*_*2*_

In the studied elite male speed skaters, the mean SmO_2baseline_ value in the VL was 64.4%. Similar results in the same muscle, using the Moxy device, were obtained in recent studies by Rębiś et al. (2024) in well-trained male speed skaters (64.3%) and Reinpõld et al. (2024) in elite male junior cyclists (63.3%), although the same authors observed a lower SmO_2baseline_ value in the group of elite male senior cyclists (57.7%). On the other hand, according to a systematic review, the observed mean resting SmO_2_ values in the VL muscle in physically active, healthy participants were higher, ranging from 68.1 to 77.9%. However, the data in this review came only from strength-trained participants and from 2 different NIRS devices (Moxy and PortaMon) (Miranda-Fuentes et al. 2021).

Studies conducted among patients with varying amounts of haemoglobin indicate that the initial values of SmO_2_ depend on its amount, but, importantly, only in people with anaemia (Crispin 2020). Noting that our sample consisted solely of healthy participants (without iron deficiency anaemia or latent iron deficiency), none of the haematological indicators correlated with the baseline value of SmO_2_, and there were no differences between the mean initial values of SmO_2_ in the subgroups with *low* and *high* tHb_mass_ (*p* = 0.94). The lack of the above-mentioned relationships in healthy people is understandable, because it is known that at rest, the blood returning to the lungs is saturated with oxygen by 75%, i.e. the body in these conditions uses only 1/4 of the oxygen contained in blood (Jensen 2009). Thus, the results of the current study confirm that in healthy people, the amount of haemoglobin in blood circulation within the reference range was able to ensure the optimal resting level of muscle oxygen saturation. Interestingly, all haematological indicators obtained using the carbon monoxide method correlated positively with the tHb_muscle_ measured by the NIRS method, and negatively with MCH, while such a relationship with tHb_muscle_ was not observed for [Hb] and Hct, probably due to the impact of plasma volume on both blood indices (Calbet et al. 2006; Schmidt and Prommer 2008; Malczewska-Lenczowska et al. 2013).

In summary, the haematological status assessed by commonly determined blood count ([Hb], Hct, MCH) and tHb_mass_ together with intravascular volume indices (PV, BV and RCV) had no effect on SmO_2baseline_. However, the results of the study suggest an effect of the haematological status on the local availability of haemoglobin in muscles (tHb_muscle_) in resting conditions, but only assessed by indices of the CO rebreathing method (BV and PV).

### Physical effort: descent phase

The applied laboratory 2-min time trial (TT) elicited a rapid muscle desaturation during the initial (descent) phase of exercise, occurring within approximately the first 20 s (descent phase). A similar decrease in muscle oxygen saturation during the first 20 s of a 3-min cycling test at a constant cadence (90 rpm) and power output (400 W) was also reported in another study speed skaters (Hesford et al. 2013). A potential objective indicator of exercise-induced changes in SmO_2_ is ΔSmO_2_. In the present study the ΔSmO_2_ recorded during the 2-min TT averaged 68.8 ± 5.5%. For comparison, in another study involving male elite speed skaters, the ΔSmO_2_ values observed for the vastus lateralis muscle (calculated based on Moxy measurements) were lower (52.0 ± 11.0%). However, the test protocol consisted of a graded intensity exercise without a prior warm-up (Rębiś et al. 2024). The relatively high ΔSmO_2_ observed in the present study may be attributed to several factors. Most importantly, the preceding warm-up likely elevated the maximal SmO_2_ value at the onset of exercise (Myers, 2019), and this value was included in the ΔSmO_2_ calculation. Additionally, existing evidence suggests that post-training improvements in exercise adaptation may be associated with an enhanced ability to perform at lower SmO_2_ levels (Paquette et al. 2020). The athletes examined in our study belonged to the elite level and demonstrated a high level of training during the testing period (August–September).

The significant decrease in muscle oxygen saturation during the descent phase of exercise was accompanied by only a slight increase in local microvascular blood volume (tHb_muscle_) despite the presumed vasodilation. It suggests that, at the onset of exercise, local oxygen consumption clearly exceeded the local capillary oxygen diffusion capacity (Korthuis 2011). To our best knowledge, this is the first study to evaluate muscle oxygen saturation responses during dynamic supramaximal efforts lasting approximately 2 min. The absence of analogous or similar studies in terms of exercise intensity and duration precludes direct comparison of our findings. During exercise, the increase in blood flow begins immediately following the onset of physical activity due to mechanical interactions between contracting and relaxing muscles and blood vessels (the muscle pump mechanism) (Joyner and Proctor 1999). This is subsequently followed by vasodilation mediated by local metabolic factors such as adenosine, nitric oxide (NO), or ATP released from red blood cells. Joyner and Casey (2015) emphasized that vasodilation requires time and typically occurs with a delay of 5–20 s after the onset of muscle contraction, which is consistent with our findings (Fig. 3).

The analysis of the entire group's data revealed that none of the haematological parameters had a significant effect on the SmO_2_ or tHb_muscle_ responses. However, when the skaters were divided into *low* and *high* tHb_mass_ groups, the study revealed that at the beginning of the 2-min TT, the oxygen saturation kinetics of the VL differed between them. It was confirmed by regression analysis which demonstrated a statistically significant, progressive increase in the SmO_2_ difference between both groups over time (*p* < 0.05) (Table 3). Moreover, participants with higher tHb_mass_ exhibited an earlier onset of SmO_2_ decline. What is more, also rel.PV and rel.BV were explanatory indices of SmO_2_ variability during the descent phase of the 2-min exercise; however, both parameters exhibited slightly weaker fits to the measurement data than relative tHb_mass_, as evidenced by marginally higher values of – 2 log likelihood (– 2LL). From the perspective of exercise physiology, the differing SmO₂ kinetics observed in the initial phase of a time trial (TT) appear unjustified, as no consistent association has been established between oxygen transport in the bloodstream and muscle oxygenation during the first few seconds of high-intensity exercise. In this early phase, the body relies primarily on immediate energy sources (Baker et al. 2010). Specifically, stored adenosine triphosphate (ATP) and phosphocreatine (PCr) support activity via the phosphagen system, enabling maximal efforts for approximately 10 s (Baker et al. 2010; Dunst et al. 2023). Only after this brief anaerobic period does aerobic metabolism begin to contribute more substantially—at which point oxygen transport may start to play a more significant role (Calbet et al. 2015; Baker et al. 2010). Moreover, Dunst et al. (2023) demonstrated that at the very onset of maximal exertion, muscle oxygen desaturation occurs independently of the rate of oxygen delivery, with the decline in SmO₂ positively correlating with phosphocreatine (PCr) dephosphorylation. Therefore, another explanation appears more likely here. It is known that high Hb_mass_, PV, and BV are typically associated with superior endurance performance (Schmidt and Prommer 2008). In our study, these indicators were also positively correlated with power output during the time trial, which is consistent with findings from other studies (Sitkowski et al. 2023; Montero et al. 2015; Heinicke et al. 2001). The higher mechanical power observed in speed skaters may have resulted in faster and greater muscle deoxygenation. The concept that greater mechanical work accelerates and amplifies muscle deoxygenation—due to rapid oxygen extraction occurring before oxygen delivery can catch up—is consistent with evidence showing that muscle oxygenation decreases quickly with rising power output, even in the early stages of exercise (Zafeiridis et al. 2015; Rębiś et al. 2024). This suggests that oxygen-carrying capacity could contribute to a higher fitness level and mechanical power output in general, and thus indirectly affect oxygenation kinetics during such an early phase of exercise. However, it remains uncertain why this association was not observed in the later stages of the effort.

Interestingly, the present study did not identify any blood count indicator that explained SmO_2_ variability during this exercise phase or correlated with rel.P_mean_. This is likely due to fluctuations in plasma volume, which cause haematological indices to not fully reflect the actual haematological status (Malczewska-Lenczowska et al. 2013; Otto et al. 2017; Vinje et al. 2024). Consequently, these indices show weak or no correlations with physical performance indicators (Schmidt and Prommer 2008), and, as demonstrated by our study, also with SmO_2_.

### Physical effort: plateau phase

After the first phase of rapid decrease in SmO_2_, for the remaining 2 min of effort performed with supramaximal power, oxygen saturation of VL remained at a low and relatively constant level (plateau phase). Similarly, local muscle blood flow (based on tHb_muscle_) after a slight increase at the beginning of exercise was stable in the plateau phase. The results of SmO_2_ and tHb_muscle_ during this part of the effort suggest the development of a balance between muscle oxygen extraction and its delivery by the microvascular system. However, the lack of similar studies in conditions of dynamic, continuous and supramaximal efforts does not allow for comparison of our results in this phase of effort.

Nevertheless, the group with higher tHb_mass_ was characterized by a greater volume of both plasma (56.3 ml/kg vs. 50.4 ml/kg in the low group, p < 0.001) and total blood volume (94.1 ml/kg vs. 83.2 ml/kg in the low group, p < 0.001), which likely resulted from earlier training adaptations (Schmidt and Prommer 2008), rather than being associated with the performed test. Additionally, the higher mean rel. P observed in this group (6.63 ± 0.51 W/kg vs. 6.34 ± 0.56 W/kg in the low group, p < 0.05), along with its significant correlations with BV, PV (p < 0.001) and tHb_mass_ (p < 0.05), suggests that intravascular volume indices within the large vessel system may contribute to the power generated during the time trial.

### Post-exercise—recovery period

The available NIRS studies indicate that the muscle reoxygenation rate following dynamic exercise is an important determinant of both muscle performance and training status (Buchheit and Ufland 2011; Bieuzen et al. 2014; McLean et al. 2016; Rębiś et al. 2024). Although haemoglobin, by enabling effective oxygen transport to tissues, plays a pivotal role in the recovery process (Fukushima et al. 2019), our results were not able to confirm this. Regression analysis conducted for the recovery phase showed that the association between SmO_2_ response and haemoglobin content only approached statistical significance (*p* = 0.052) (Table 2). Similarly, in the entire group, borderline significant correlations were observed between t½SmO_2_ and both relative tHb_mass_ (*p* = 0.06) and RCV (*p* = 0.05). Moreover, although significant negative correlations were found between relative PV and relative BV and t½SmO_2_ (Tab.2)—indicating that greater plasma and blood volumes were associated with shorter muscle reoxygenation times—the strength of these correlations was only moderate (explained no more than 30% of the total variance), with relatively low statistical significance (p < 0.05). Importantly, no correlations were observed during recovery between t½SmO_2_ and red blood cell parameters ([Hb], Hct, MCH) (Fig. 2), and no significant associations of these variables during the overall recovery period were detected (Table 2). Further research is required to more comprehensively elucidate the role of haematological parameters in the recovery process.

Of note, the post-exercise reoxygenation rate of VL muscle was approximately twice as slow as the deoxygenation rate during the initial phase of exercise (+ 2.03 ± 0.62%/s vs. – 4.2 ± 1.06%/s, respectively). However, these findings should be interpreted with caution due to the intercept characteristics (only one waveform was linearly approximated from time = 0).

### Strengths, limitations, and further research

The primary strength of this study lies in its novelty. To our knowledge, this is the first study to examine the relationships between haematological status (assessed using two different methods) and local muscle oxygen saturation, as well as the first to analyse changes in SmO_2_ and tHb_muscle_ during a 2-min time trial. However, noteworthy limitations should be acknowledged. First, the study employed a cross-sectional design, which limits the ability to draw causal inferences. As the results represent single time points, they may not account for temporal changes or the dynamic nature of haematological changes, training adaptations, and athletic performance. Next, although a unique cohort of highly-trained athletes was recruited, the included sample was relatively small.

Finally, only male athletes were included in the study, which precluded the evaluation of potential sex-based differences in the investigated variables. Future research should explore these relationships under different training conditions, such as hypoxic exposure or in athletes with documented iron deficiency. Moreover, research on females and populations of different fitness status seems warranted.

## Conclusions

In healthy, well-trained athletes, haematological status did not appear to influence resting muscle oxygen saturation (SmO_2_), although pre-exercise tHb_muscle_ may depend on relative plasma and blood volume.

The observed relationship between haematological status and SmO_2_ at the onset of exercise is insufficient to conclusively demonstrate an influence on SmO₂ kinetics throughout the entire effort.

Muscle reoxygenation dynamics may be associated with haematological parameters but only derived from the carbon monoxide rebreathing method.

Finally, the observed SmO₂ response pattern suggests that muscle deoxygenation at the onset of exercise may occur more rapidly than reoxygenation during recovery.

## Data Availability

The datasets generated during and/or analysed during the current study are available from the corresponding author on reasonable request.

## Abbreviations

ATT: Skinfold thickness in the vastus lateralis (VL) area
BMI: Body mass index
BV: Blood volume
[Hb]: Blood haemoglobin concentration
Hct: Haematocrit
La3min: Blood lactate concentration at 3 min after exercise test
MCH: Mean corpuscular haemoglobin in erythrocytes
MCV: Mean corpuscular volume (red cell volume)
NIRS: Near-infrared spectroscopy
*P*_mean_: Mean power
PV: Plasma volume
RBC: Red blood cells
tHb_mass_: Total haemoglobin mass
rel.tHb_mass_.high: The group with rel.tHb_mass_ above the median value
rel.tHb_mass_.low: The group with rel. tHb_mass_ below the median value
SmO_2_: Muscle oxygen saturation
SmO_2baseline_: Local muscle oxygen saturation before exercise (3 min after warm-up)
SmO_2max_: Maximum local muscle oxygen saturation during the 2-min time trial
SmO_2min_: Minimum local muscle oxygen saturation during the 2-min time trial
ΔSmO_2_: Difference between maximum and minimum muscle oxygen saturation during the time trial
t½SmO_2_: Half-time of muscle reoxygenation
tHb_muscle_: Local haemoglobin concentration in the muscle
tHb_muscle_.cent: Person-mean-centred value of tHb_muscle_ in each phase
